# Burden and satisfaction with social support in families with a history of problematic substance use or dementia – a comparison

**DOI:** 10.1186/s40359-024-01940-1

**Published:** 2024-08-21

**Authors:** Renate Soellner, Christine Hofheinz

**Affiliations:** Institute of Psychology, University of Hildesheim, Universitätsplatz 1, 31141 Hildesheim, Germany

**Keywords:** Social support, Burden, Dementia, Problematic substance use, Family members

## Abstract

**Background:**

Living in a family with a history of problematic substance use or dementia is a major stressful experience for family members and results often in an impaired health condition. Seeking and receiving social support has been emphasized as a major resource while coping with this stress. However, especially family members of people with problematic substance use often refrain from help-seeking. This paper examines whether (1) family members of problematic substance users are more physically and psychologically distressed than caregivers of people with dementia, and (2) whether and to what extent differences are mediated by satisfaction with perceived professional and private social support.

**Methods:**

Two samples of family members of people with a chronic disease (problematic substance use (*n* = 221), dementia (*n* = 322)) completed self-administered questionnaires on burden, quality of life and social support. Physical distress was assessed using the Giessen Subjective Complaints List, psychological distress using the Center for Epidemiological Studies Depression Scale and anxiety symptoms using the Hospital Anxiety and Depression Scale. Quality of life was measured using the WHOQOL-BREF, and satisfaction with professional and private social support was assessed using a visual analog scale. Multivariate analyses of variance were performed to compare the two groups on the means of (1) burden ratings and (2) QoL dimensions and followed up by discriminant analyses to explore meaningful variables according to group differences. Parallel mediation analyses were performed to test the mediators private and professional support.

**Results:**

Family members of problematic substance users score higher on the burden indicators, while they score lower with regard to the quality of life dimensions than caregivers of people with dementia. The difference in burden is mainly explained by the higher scores for exhaustion, stomach discomfort and depression of family members of problematic substance users. Caregivers of people with dementia reported greater satisfaction with perceived social support, either private or professional. Satisfaction with private support was shown to be more important in mediating the impact of stress.

**Conclusions:**

Family members of people with problematic substance use are in great need of tailored support programs and should be explicitly targeted.

## Background

Family members of persons with a chronic disease, often requiring intensive care, generally have poorer health [1]. For example, caregivers of people with dementia report an increased prevalence of anxiety and depressive symptoms, as well as poorer physical health and quality of life [2, 3]. Impaired caregiver health has also been reported after stroke and for people with family members with mental disease [4, 5]. Among these, relatives of people with substance use problems (SUP) are a special group. Living together with someone who misuses alcohol or drugs places a heavy burden on family members or friends [6–8]. Family members describe ongoing insecure living conditions, conflicts over money and personal possessions, and a relationship with the substance-using person that becomes uncomfortable and sometimes aggressive [9]. They show increased symptoms of depression, anxiety and stress [10], reduced quality of life, and higher levels of physical (exhaustion, gastrointestinal disorders) and psychological (somatization, uncertainty in social contacts) distress compared to the general population [8].

In order to better describe and understand the situation faced by family members of substance users Orford and colleagues have developed a stress-strain-coping support model [11]. They emphasize the role of the quality of social support, which is not simply measured by the number of people available, but by how well the support works to adequately address the problem. According to this model, for family members of problematic substance users, social support (e.g. emotional, informational or practical) is particularly important for coping, but often fails [9]. Because seeking and receiving social support plays an important role in coping with stress, this is a major health risk factor [12, 13]. Referring to the concept of social support, it has been repeatedly suggested that perceived and received social support, which were found to be only moderately correlated, should be distinguished [13, 14]. Perceived social support refers to how persons rate how accessible and valuable their potential support is, while received support refers to the amount of support they actually receive. Perceived social support was found to be more predictive of mental health, such as depression [15], while received support was only marginally predictive [16]. Among caregivers of people with dementia or older adults, at least two studies have found that social support mediates the relationship between resilience and stress [17, 18]. A meta-analysis of caregivers of adults and older adults found a moderate, negative association of perceived social support with subjective distress while the negative association of received support with subjective distress was only small [19]. Social support through professional or private help promotes mental as well as physical health [20, 21]. In particular, informal social support was found to be more important than professional support [22]. For example, in a meta-analysis of 148 studies, Holt-Lundstad and colleagues [23] showed that both social integration and social support, in the form of perceived support from others, were associated with reduced mortality, even exceeding the effects of behavioral factors such as physical activity or excessive drinking.

Family members of people with SUP often refrain from seeking professional services in the first place. For example, 58% of the interviewed relatives of substance misusers in Brazil waited an average of 2.6 years before actively seeking professional help [24]. Most of them thought that substance misuse was a temporary problem (40.6%) or did not know what kind of help they needed (29.7%). About 10% reported that they had tried to hide the problem. In addition, family members often believe that the problem should be dealt with within the family [6].Specifically, they perceive professionals as lacking in knowledge, awareness or empathy and feel excluded from treatment.

One possible explanation for this lack of social support for family members lies in the stigma experienced by people with SUP [25–27]. In a study on the factor structure of public stigma of substance use disorders (SUD), 337 participants rated how they think the public would believe certain statements on “addicts”. A four-factor solution was found for stereotypes including themes like reckless (unpredictable, uncontrollable), unreliable (weak, hopeless, selfishly), inadequate (worthless, lazy, to blame for their own problems), and threat (they are seen as cheaters and liars) [28]. Furthermore, people were expected to form prejudices, covering emotional reactions like anger (leading to hate and resentment), pity (because they do not know how to help), and dread (being afraid or wary of people with SUP) [28]. The process of being stigmatized has been widely reported for people with mental illness in general [29, 30]. In a representative sample in the USA, people with various mental disorders were assessed for help-seeking behavior [31]. People with SUPs were found to be less likely to seek help than people with mood disorders in general. The topic is still relevant today, especially in the light of the alarmingly rising rates of overdoses and opioid and stimulant consumption within the last years in the United States. Krendl and Perry [26] recently provided a comprehensive overview on research on stigma on SUD and non-substance related mental illness. They conclude, that people with SUD are generally viewed as more dangerous than people with mental illness and that stigma is a barrier not only to seeking treatment but also predicts negative attitudes toward treatment seeking.

Stigma has been shown to affect not only the person with mental illness but also their families [32, 33], the so called ‘courtesy stigma’ [34]. This is also evident, for caregivers of people with dementia, who report feeling stigmatized [35]. As a consequence, caregivers and other family members refrain from seeking help due to fear of being stigmatized [9, 36, 37] or self-stigmatize. Self-stigma, in turn, is defined as endorsing negative stereotypes about oneself [38]. Family caregivers of people with dementia, for example, self-stigmatize ‘being a neglectful caregiver’ or ‘receiving a punishment from God’ which results in isolation and not seeking help [39]. However, as dementia and its consequences have received increasing attention in policy and public discourse over the past two decades, dementia has been somewhat de-tabooized. Support for people with dementia and their families, such as Alzheimer’s Society family groups and tailored interventions such as TeleTAnDem [40], has increased, and caregivers of people with dementia are more likely to receive and actively seek formal or informal social support.

While help-seeking per se is stigmatized and leads to reduced help-seeking behavior [41], Clement and colleagues [42] found a median association between stigma and help-seeking of d=-0.27 in a systematic review of the impact of mental health stigma on the formal service use, which equals a small effect size [43]. Increasing stigmatization is therefore accompanied by less help-seeking behavior. Self-stigmatization and anticipated discrimination when seeking help were most frequently associated with reduced help-seeking.

In summary, literature on stigma and mental illness suggests that family members of people with SUP are particularly affected by both public stigma and internalized self-stigma, resulting in less help-seeking behavior and, hence, less social support. However, the quality of social support, particularly informal perceived social support, is very important in reducing the burden of the family situation. Given these differences in stigma for family members of people with SUP on the one and caregivers of people with dementia on the other hand, we expect greater burden and poorer mental and physical health condition for family members of people with SUP. Furthermore, we expect, that caregivers for people with dementia will be more satisfied with the social support they perceive compared to family members of people with SUP, which mediates this complex relationship.

In this paper we will examine whether (1) family members of problematic substance users are more physically and psychologically distressed than caregivers of people with dementia, and (2) whether and to what extent differences in physical and psychological distress are mediated by satisfaction with perceived professional and private social support.

## Methods

### Participants and procedure

The sample for this paper is taken from two research projects with family members of people with a chronic disease: (1) a participatory research project with relatives of people with SUP (*n* = 221) in 2015-2017 [7] and (2) a controlled cognitive behavioral intervention study with caregivers of people with dementia (*n* = 322) taking place in the years 2013–2015 [44]. Both samples were convenience samples and were gathered via newspaper advertisements, self-help groups or e.g. the Alzheimer Society. In this paper the baseline measures of both samples are compared. Thus, we do not assume any interaction of the answering behavior with the treatment (two-arm controlled intervention study versus participatory approach). Family members of persons with SUP (FM group) were mainly interviewed online from January to 2016 to August 2016, while the questionnaires for caregivers of people with dementia (CG group) were collected by paper and pencil between September 2012 to November 2013. Recruitment for both projects used a variety of publicity relations methods: printed information materials available at cooperation partners (clinics, practices, home support services), editorial articles in regional and national newspapers, television and radio interviews, project websites, online newsletters and postings in online groups. To be included, participants in the FM group had to identify themselves as relatives of a problematic substance user. A medical diagnosis of an addiction was not required and there were no exclusion criteria. Inclusion criteria for the CG group were somewhat stricter: Participants had to be the main person responsible for the care of the person with dementia, and the person with dementia must had to have at least low-grade dementia according to medical diagnosis. Exclusion criteria were that the caregiver was receiving ongoing psychotherapeutic treatment, had a serious physical illness or medically diagnosed psychiatric disorder, and that the person with dementia was institutionalized or planned to be institutionalized in the next 6 months.

Both research projects were approved by the Ethics Committee of the respective Universities (University of Hildesheim, Friedrich-Schiller‐University Jena (3453‐05/12)). All participants provided written informed consent.

### Measures

#### Burden

Physical distress was assessed using the exhaustion and stomach discomfort subscales of the short version of the *Giessen Subjective Complaints List* (*Gießener Beschwerdebogen*,* GBB-24*) [45]. Participants had to indicate how much each of the six complaints per subscale bothered them (0 = not at all, 4 = strongly). Cronbach’s alpha was 0.90 for exhaustion and 0.74 for stomach discomfort for the caregivers and 0.87 for exhaustion and 0.83 for stomach discomfort for the family members of people with SUP. Psychological distress was assessed by measuring symptoms of depression and anxiety. Depressive symptoms were assessed using the German version of *Center for Epidemiological Studies - Depression Scale* (*Allgemeine Depressionsskala*, *ADS*)) [46]. The ADS measures how often 20 symptoms applied to the participant in the past week (0 = rarely/less than 1 day, 3 = mostly/5–7 days). Cronbach’s alpha was 0.89 for the caregivers [3] and 0.91 for family members of persons with SUP. Anxiety symptoms were derived from the anxiety subscale of the *Hospital Anxiety and Depression Scale* (*HADS*) [47] (German version [48]). Participants were asked to rate how often they had experienced seven symptoms in the past week. Cronbach’s alpha was 0.81 for the caregivers and 0.82 for family members.

#### Quality of Life (QoL)

QoL was measured using the *WHOQOL-BREF* (WHO, 1996; German version: [49]) with the four domains *physical* (seven items), *psychological* (six items), *environmental* (eight items), and *social relationships QoL* (three items). Participants rated their satisfaction with the quality in each domain over the last 2 weeks on a 4-point Likert scale. Internal consistency, as measured by Cronbach’s Alpha, ranged from 0.58 (social relationships), 0.72 (environment), 0.81 (psychological health) to 0.83 (physical health) for the caregivers [50]. A similar pattern emerged for the family members of people with SUP, again with internal consistency worst for social relationships (0.57), but in the good range for physical health (0.79), environment (0.81) and psychological health (0.87). Raw data for each domain were transformed into summed scores ranging from 0 = not at all satisfied to 100 = very satisfied.

#### Satisfaction with social support

Satisfaction with professional, personal and emotional social support was measured using a one-item visual analog scale for each facet. Participants had to indicate their satisfaction with each type of support (from 0 = not at all satisfied to 100 = very satisfied). These scales were developed in study 2 along with several other one-point item measures relating to burden of care and emotional wellbeing [44]. For this paper the personal and emotional support facets were aggregated into a new variable “satisfaction with private support”, with the higher value of the two original variables.

### Statistical analysis

Chi-square and t-tests in SPSS 27 were used to assess differences between the FM and the CG groups on socio-demographic characteristics. Two multivariate analyses of variance (MANOVA) were performed to compare the two groups on the means of (1) the four burden assessments and (2) the four QoL domains. MANOVA assumptions regarding multivariate normality and homogeneity of variance covariance matrices were tested using the BOX-M-Test. Given different sample sizes, with larger variances and covariances of the smaller sample size, a significant Box-M-Test may be too liberal and should be treated with caution [51]. However, if the assumption of multivariate normality is met, the assumption of homogeneity of variances can be safely assumed [52]. Therefore, to check for multivariate normality, the Mahalanobis distance was additionally plotted against the Chi-square [53]. The MANOVAs were followed up by discriminant analyses to explore meaningful variables according to group differences. Mediation analyses were conducted by using the PROCESS macro for SPSS [54]. Variables that were found to be significantly different between the two groups in the discriminant analyses (*r* > .5) were included as outcomes in the mediation models. Satisfaction with professional and private support were included as mediators in a parallel mediation analysis.

## Results

### Sample characteristics

Sociodemographic characteristics are shown in Table 1. Participants in both groups were predominantly female (FM = 91.2%; CG = 80.1%). Caregivers of people with dementia were significantly older than family members of persons with SUP (*t*_(537)_ = -17.85, *p* < .001). While the gender ratio among the ill relatives in the CG group is relatively balanced (51.2% female), in the FM group it is predominantly men who are affected by the substance use problem (19.6% female). Regarding the most common substance used in the FM group was alcohol (71.9%), followed by cannabis (30.3%) and other illicit drugs (26.2%). The most common type of dementia in the CG group was Alzheimer’s disease (44.4%), followed by vascular dementia (10.3%).

**Table 1:** Sociodemographic characteristics of both groups

	FM *n* = 221	CG *n* = 322	test statistic	*p*
	*n* (%) *		χ^2^	
**Gender (female)**	198 (91.2)	258 (80.1)	13.33	< 0.001
**Gender of ill person (female)**	42 (19.6)	165 (51.2)	53.20	< 0.001
**Relation to ill person**				
partner	127 (59.6)	189 (58.7)	40.21	< 0.001
parent/ child/ sibling	55 (25.8)	128 (39.8)		
relative	10 (4.7)	3 (0.9)		
friend	21 (9.9)	2 (0.6)		
**Shared household**	89 (42.2)	254 (79.6)	77.98	< 0.001
		***M (SD)***	***t***	
**Age**	44.75 (13.95)	64.06 (11.09)	−17.85	< 0.001

Note: FM = family members of problematic substance users; CG = caregivers of people with dementia; * due to missing values percentages might not exactly apply to column n

### Differences in Burden and QoL

Using Pillai’s trace the two MANOVAs on four indicators of burden and four dimensions of QoL showed both significant differences between the two groups of relatives (*V* = 0.077, *F*(4,514) = 10.65, *p* < .001; *V* = 0.034, *F*(4,507) = 4.04, *p =* .002; Table 2). Family members of problematic substance users score higher in the indicators of burden while they score lower with regard to the QoL dimensions than caregivers of people with dementia.

**Table 2:** Mean values (standard deviation) for outcome measures and MANOVA results

	Mean (SD)		MANOVA			
	FM	CG	*V*	*F*	*p*	η2*
**Burden**			0.077	10.65	< 0.001	0.077
Exhaustion (GBB-24)	11.31 (5.60)	9.20 (5.60)				
Stomach discomfort (GBB-24)	5.28 (4.98)	3.12 (3.41)				
Depression (ADS)	25.68 (12.18)	22.35 (9.75)				
Anxiety (HADS)	10.42 (4.47)	9.60 (4.18)				
**Quality of life**			0.034	4.40	0.002	0.034
physical	60.17 (18.32)	63.27 (18.26)				
psychological	48.70 (20.62)	55.46 (17.62)				
environmental	68.47 (17.38)	70.38 (13.94)				
social	46.05 (20.69)	48.36 (19.96)				

Note: FM = family members of persons with SUP; CG = caregivers of people with dementia; GBB-24 = Giessen Subjective Complaints List; ADS = Center for Epidemiological Studies - Depression Scale; HADS = Hospital Anxiety and Depression Scale* *η2* and *V* are identical

Following up the MANOVA results with a discriminant analysis for the indicators of burden one significant discriminant function emerged (*Λ* = 0.92, *χ*^*2*^(4) = 41.02, *p* < .001, canonical *R*^*2*=^0.076). The correlation between the independent variables and the discriminant function showed that exhaustion loaded the highest (*r* = .894), stomach discomfort and depression loaded in a middle range (*r* = .636, *r* = .524,), and that anxiety was less discriminant (*r* = .325). Hence, the difference in burden between both groups is predominantly explained by the higher exhaustion, stomach discomfort, and depression values of the family members of problematic substance users. Also, the discriminant analysis for the QoL dimensions turned out into one significant discriminant function (*Λ* = 0.97, *χ*^*2*^(4)17.35, *p* = .002, canonical *R*^*2*^ = 0.033). Here, only psychological QoL discriminated strongly between family members of substance users and caregivers of people with dementia (*r* = .935), while all other dimensions correlated with the discriminant function less than 0.5. Again, family members of people with SUP show a poorer psychological health condition compared to caregivers of people with dementia.

### Mediation analysis

**Fig. 1:**
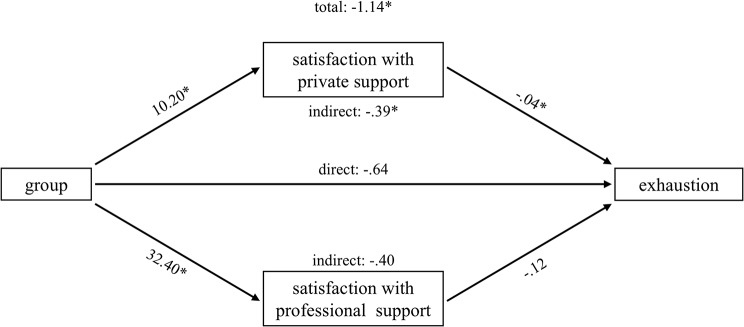
Parallel mediation model: exhaustion

**Fig. 2:**
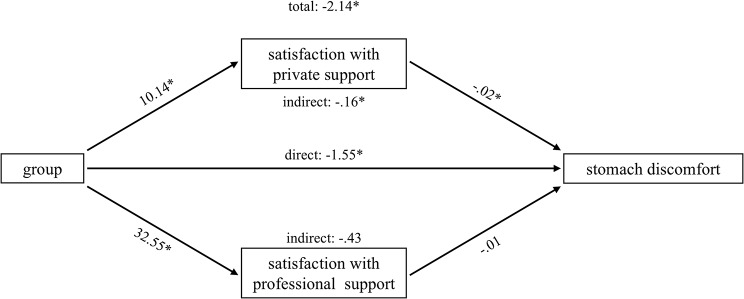
Parallel mediation model: stomach discomfort

**Fig. 3:**
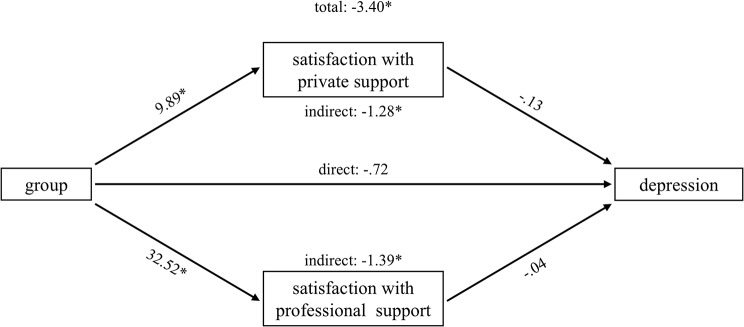
Parallel mediation model: depression

**Fig. 4:**
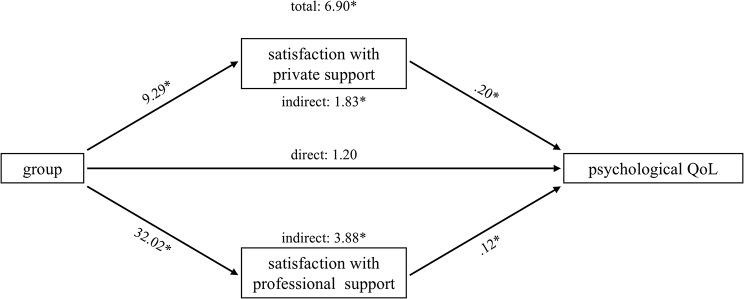
Parallel mediation model: psychological QoL

Parallel mediation analyses were conducted for the four outcomes that correlated at least 0.5 with the discriminant function: exhaustion, stomach discomfort, depression and psychological QoL. The results for each path are displayed in Table 3. Total, direct and indirect effects are shown in Table 4. Graphs of the various effects are provided in Figs. 1–4.

In general, caregivers of people with dementia reported being more satisfied with perceived social support regarding both, private and professional support, than family members of people with SUP, while this difference was obviously more pronounced for professional support than for private support (e.g. exhaustion: b = 32,41 vs. b = 10,25). The direct effects of the mediators on the outcome variables were mixed. Satisfaction with private support was negatively associated with exhaustion and stomach discomfort, and positively associated with psychological QoL. Satisfaction with professional support only predicted psychological QoL positively. There was no direct significant association between any of the satisfaction with social support measures and depression.

However, both mediators show specific indirect effects for depression (private support: -1,2857, [CI: -2,1754 - -,5182]; professional support: -1,3924, [CI: -2,8111 - -,0821]) and psychological QoL (private support: 1,8270, [CI: ,6584-3,2417]; professional support: 3,8787, [CI: 1,4299–6,3860]). Caregivers of people with dementia are less depressed and report a higher QoL, which is mediated by satisfaction with private and professional social support. With regard to stomach discomfort and exhaustion, only satisfaction with private social support significantly mediated the effect of group membership (stomach discomfort: -,1573, [CI: -,3740 - -,0026]; exhaustion: -,3866, [CI: -,7118 - -,1354]). The FM group shows higher levels of exhaustion and stomach discomfort than the CG group, which are both mediated by satisfaction with private social support.

**Table 3:** Results of mediation analyses

Variables	M1 (private support)			M2 (professional support)			Y		
	Coefficient	*SE*	95% CI	Coefficient	*SE*	95% CI	Coefficient	*SE*	95% CI
							*exhaustion*		
Group^a^	10,2525	2,9322	**4**,**4878– 16**,**0172**	32,4166	2,5112	**27**,**4795– 37**,**3537**	-,6435	,6618	-1,9446–,6576
M1							-,0377	,0098	**-**,**0570–** **-**,**0185**
M2							-,0124	,0114	-,0349– ,0100
Constant							13,7078	,7397	12,2536– 15,1620
Model Fit	R^2^=,0613, F(3,393) = 8,5536, *p* < .001								
							*stomach discomfort*		
Group*	10,1402	2,9496	**4**,**4878–15**,**9393**	32,5593	2,5196	**27**,**6056–37**,**5130**	-1,5534	,5290	**-2**,**5935–** **-**,**5133**
M1							-,0155	,0078	**-**,**0308–**,**0002**
M2							-,0131	,0091	-,0311–,0048
Constant							6,7414	,5938	5,5739–7,9088
Model Fit	*R*^*2*^=,0742, *F*(3,387) = 10,3390, *p* < .001								
							*depression*		
Group^a^	9,8914	2,9880	**4**,**0164–15**,**7664**	32,5663	2,663	**27**,**4805–37**,**57**,**22**	-,7196	1,2452	-3,1679–1,7287
M1							-,1300	,0184	-,1661–,0939
M2							-,0428	,0214	-,0849–,0008
Constant							34,7337	1,3944	31,9920–37,4755
Model Fit	*R*^*2*^=,1638, *F*(3,382) = 25,9412, *p* < .001								
							*psychological quality of life*		
Group*	9,2997	2,9945	**3**,**4117–15**,**1878**	32,0222	2,5853	**26**,**9387–37**,**1056**	1,1982	2,1355	-3,0008–5,3972
M1							,1965	,0319	,**1338–**,**2591**
M2							,1211	,0369	**0.0485–**,**1937**
Constant							32,2489	2,4387	27,4536–37,0441
Model Fit	*R*^*2*^=,1693 *F*(3,374) = 25,4118, *p* < .001								

Note: Significant results are in bold; ^a^Group (0 = FM, 1 = CG)

**Table 4:** Results of mediation analyses. Total direct and indirect effects

Type	Effect	Coefficient	SE	95% CI lower	95% CI upper
*exhaustion*					
indirect	X = > M1 = > Y X = > M2 = > Y	-,3866 -,4031	,1487 ,3926	**-**,**7118** -1,1920	**-**,**1354** ,3535
direct	X = > Y	-,6435	,6618	-1,9446	,6576
total	X = > Y	-1,4331	,5667	**-2**,**5472**	**-**,**3190**
*stomach discomfort*					
indirect	X = > M1 = > Y X = > M2 = > Y	-,1573 -,4270	,0959 ,3166	**-**,**3740** -1,0512	**-**,**0026** ,1845
direct	X = > Y	-1,5534	,5290	**-2**,**5935**	**-**,**5133**
total	X = > Y	-2,1377	,4456	**-3**,**0137**	**-1**,**2616**
*depression*					
indirect	X = > M1 = > Y X = > M2 = > Y	-1,2857 -1,3924	,4229 ,6908	**-2**,**1754** **-2**,**8111**	**-**,**5182** **-**,**0821**
direct	X = > Y	-,7196	1,2452	-3,1679	1,7287
total	X = > Y	-3,3977	1,1270	**-5**,**6136**	**-1**,**1818**
*psychological quality of life*					
indirect	X = > M1 = > Y X = > M2 = > Y	1,8270 3,8787	,6558 1,2584	,**6548** **1**,**4299**	**3**,**2417** **6**,**3860**
direct	X = > Y	1,1982	2,1355	-3,0008	5,3972
total	X = > Y	6,9038	1,9367	**3**,**0956**	**10**,**7120**

Note: X = group (0 = FM, 1 = CG), Y = outcome, M1 = private support, M2 = professional support; significant results are in bold

## Discussion

Living in a family with a history of problematic substance use or dementia is a major stressful experience for family members and results often in an impaired health condition. Within this study family members of persons with either substance use problems or dementia were compared concerning their level of burden. It is further argued, that poorer health may be mediated by reduced help-seeking behavior and thus lower satisfaction with social support.

Caregivers of people with dementia showed less stress than family members of people with problematic substance use. In fact, the latter suffered from higher burden on all variables, while exhaustion, followed by stomach discomfort and depression, being the most distinctive. As caregivers of people with dementia have been repeatedly described as highly exhausted due to the physical and psychological demands of caring for a person with dementia [3, 55], this finding underlines the even higher burden faced by family members of people with a history of substance abuse. This is supported by recent research describing mothers of children with substance use disorders as having higher levels of burnout and depression than mothers of patients suffering from schizophrenia [56]. However, other studies report that the burden of caregivers respectively family members of these two mental illnesses is high but equivalent [57].

Regarding our second research question, we found that perceived social support did mediate the differences in psychological and physical distress but the results depended on the type of support. While satisfaction with private support mediated all group differences, satisfaction with professional support was relevant only for depression and psychological quality of life. This finding is in line with previous research, showing that people generally rely more on informal sources such as family and friends and seek less professional support, while seeking professional support is highest among those suffering from more severe conditions [58]. Again, in a qualitative study with caregivers of dementia, emotional support was identified as one of the most important aspects of caregivers’ health needs [59].

As family members of persons with SUP were less satisfied with both, private and professional social support, their mental health outcomes were worse. Given the higher level of stigmatizing experienced by people with SUP, their family members appear to be equally affected. Living with, or being close to, a problematic substance user presents specific challenges. Amongst them are uncomfortable relationships, sometimes aggressive behavior, constant worry and fear of public embarrassment, and repeatedly dashed hopes that the loved one will be abstinent [11, 57]. They lead to a range of negative emotions, such as anger, fear, guilt, shame, and hopelessness which are associated with increased levels of stress, depression and anxiety [10, 60]. These stressors as well as the high level of self-stigmatizing of family members of people with SUP [56], may have an additionally negative impact on the mental and physical health status of this group.

One possible explanation may be that family members of people with SUP are reluctant to proactively seeking help for fear of shame and guilt. Less satisfaction with existing support structures for families with a history of problematic substance use could also be seen as a consequence of the reduced help-seeking behavior of families with people with SUP. In general, while support structures for caregivers of people with dementia have been developed and become more accessible over the last decade, family members of people with SUP are still rarely specifically addressed in the support system. This is also true in the private sector, where support for dementia seemed to be much more accessible and affordable than for a family member of people with problematic substance use. In other words, doing the shopping or looking after a person with dementia seems easier and less embarrassing for the supporting person than dealing with a person who is behaving aggressively under the influence of alcohol.

However, although social factors contribute about as much to explaining life expectancy as well-known behavioral risk factors such as smoking, high alcohol consumption and physical inactivity, the importance of social factors for health is still underestimated by the public [61]. It is a societal responsibility to disseminate this knowledge and promote the development of tailored support structures for family members of people with problematic substance use. Moreover, the structural discrimination against people experiencing substance-related problems and their families finally need to be broadly addressed to change the game [62]. This includes adequate media coverage, using person-first language, i.e. speaking from persons with substance use instead of substance users [26], risk-factor reduction as well as treatment access and humanizing narratives [63].

### Strengths and limitations

This is the first paper to compare the two groups of family members, those of people with problematic substance use and those of people with dementia. Given the high and rising prevalence of these diseases, research on this topic is particularly important. The results therefore affect a large number of people. From a clinical perspective, they indicate that although both groups suffer from psychological and physiological distress, family members of people with SUP may be in particular need of more appropriate professional and private support. This means that those working in the help system should automatically think about the relatives of people with problematic substance use. Whenever a person enters the help system because of substance use problems, the relatives should also be immediately considered and provided with specific services.

There are also some limitations to note. As our sample consists of family members of people with chronic diseases, our data only represent the views of family members and not the patients themselves. We measured satisfaction with social support as a proxy for help-seeking behavior on the one hand and stigma on the other. We expected differences in distress variables to be mediated by satisfaction with perceived social support, but cannot show whether they are mediated by stigma. Future research should pick up that issue and investigate stigma and help-seeking behavior directly. However, since the results support our expectations of different levels of stress and perceived social support, our expectation of different levels of stigmatization associated with these diseases are at least plausible and not rejected.

Importantly, measurement took place mostly online for the FM group and paper and pencil with telephone support for the CG group. Therefore, we cannot rule out the possibility that the lower burden within the CG group was also due to the fact that the telephone support made the survey less anonymous when completed. The telephone support was provided by other members of the research group and the authors of this study and the respective therapists in the intervention project were not involved. Nevertheless, it is possible that some CGs found it more difficult to express their true distress in telephone contact. Although both samples were recruited differently, both were convenience samples. Therefore, the issue of specific representativeness needs to be considered. A major limitation is the age difference between the two groups, as family members of people with SUP were considerably younger than those caring for people with dementia. In addition, people with problematic substance use were predominantly male, whereas the people with dementia were equally divided between men and women. However, as problematic substance use tends to occur earlier in life than dementia, we believe that these samples are representative of the specific condition. We deliberately chose not to use matching techniques such as propensity score matching, as this would have resulted in both samples being truncated on one side of the distribution, resulting in a superficial sample. Rather, we believe that the age of the family members and the gender of those affected seem to be inherent characteristics of the sample of question. Although the data are from before the pandemic, we do not expect any changes in the interplay between stigma, satisfaction with social support and health outcomes. Furthermore, there is no evidence that the structures of support systems and attitudes towards mental illness, particularly substance use problems, have changed. Lastly, based on cross-sectional data, mediation analyses do not necessarily imply causality. It is thus also possible, that a higher burden could lead to lower satisfaction with social support.

## Conclusions

People with problematic substance use and their family members should be addressed at a societal level. In addition to educational programs broad structural measures are needed to initiate social change and to reduce structural discrimination. It is long overdue that people with SUP and their families are assessed and treated in the same way as people with other mental and non-mental diseases. More tailored programs need to be developed to support family members of people with a history of substance abuse. Emphasis should be placed on overcoming self-stigma, coupled with proactively seeking and accepting social support. Access to the support system should be broadened according to their specific situation. This can be one component in a necessary package of measures towards a more inclusive and solidary society, where everyone has their place.

## Data Availability

The datasets generated and/or analysed during the current study are not publicly available due to the Ethics approval and the data contract for both projects.

## Abbreviations

SUP: Substance use problem
SUD: Substance use disorders
QoL: Quality of life
FM: Family Members
CG: Caregivers
